# Immigration, perceived discrimination and mental health: evidence from Venezuelan population living in Peru

**DOI:** 10.1186/s12992-020-00655-3

**Published:** 2021-01-07

**Authors:** Benoît Mougenot, Elard Amaya, Edward Mezones-Holguin, Alfonso J. Rodriguez-Morales, Báltica Cabieses

**Affiliations:** 1 Centro de Excelencia en Investigaciones Económicas y Sociales en Salud, Universidad San Ignacio de Loyola, Lima, Peru; 2 Facultad de Ciencias Empresariales, Universidad San Ignacio de Loyola, Lima, Peru; 3 Epi-gnosis Solutios, Piura, Peru; 4 Public Health and Infection Research Group, Faculty of Health Sciences, Universidad Tecnológica de Pereira, Pereira, Risaralda Colombia; 5 Grupo de Investigación Biomedicina, Faculty of Medicine, Fundación Universitaria Autónoma de las Américas, Pereira, Risaralda Colombia; 6 Instituto de Ciencias e Innovación en Medicina (ICIM), Facultad de Medicina Clínica Alemana, Universidad del Desarrollo, Santiago, Chile

**Keywords:** Migration, Mental health, Discrimination, Venezuelans, Peru

## Abstract

**Background:**

The association between international migration and mental health is conditioned to several factors, and discrimination may play a significant role. Currently, Peru is one of the principal Venezuelan migrant-receiving countries in Latin America. There are around one million Venezuelan refugees and migrants in the country. This study evaluates the association between self-perceived discrimination and mental health problems in Venezuelan population living in Peru.

**Method:**

We analyzed data from the Venezuelan Population Residing in Peru Survey 2018, a nationally representative urban sample aimed at collecting information on several dimensions of Venezuelan population wellbeing. We applied logistic regression models to assess the association between self-perceived discrimination and mental health problems. Moreover, we applied the propensity score matching method as a robustness check of our results.

**Results:**

Of 9487 Venezuelans surveyed, 6806 included complete information. From this sample, 6.3% reported mental health problems related to fear, anger, anxiety, or stress. Logistic regression models showed that Venezuelans who perceived being discriminated against had 2.4 higher odds of presenting mental health problems than their non-discriminated counterparts. Moreover, propensity score matching models showed that Venezuelans who perceived being discriminated against increased by 3.5 percentage points their probability of presenting mental health problems compared to their non-discriminated counterparts.

**Conclusions:**

There is evidence that self-perceived discrimination is associated with mental health deterioration in Venezuelan migrants living in Peru. Our findings are relevant in the current geopolitical context and could be useful in the decision making processes in international health.

**Supplementary Information:**

The online version contains supplementary material available at 10.1186/s12992-020-00655-3.

## Background

International migration is a complex phenomenon that involves a multiplicity of economic, social and security contents given an increasingly interconnected world. The lives of international migrants are challenging and several social factors can affect their wellbeing during transit and settlement [1, 2]. Many migrants labor in informal jobs associated with human rights violations, undignified wages, and exploitation; and many live in low quality and overcrowded households [2, 3]. They have limited healthcare access, and many of them fear using the healthcare system when required [1]. They also experience multiple types of discrimination when trying to integrate into the host society [4, 5]. Consequently, the study of discrimination as a factor that affects the health of international migrants is a highly important matter.

Since the 2000s, international migration flows in Latin America have shown changes in direction and intensity. Currently, the “Venezuelan phenomenon” has brought essential repercussions in the region. The Venezuelan migration process is based on three phases, shifting from a migrant-receiving to a migrant-sending country [6]. The first phase began in 2000 after Hugo Chávez won the presidential election and implemented the “Socialism of the Twenty-First Century”. Venezuelans from the upper classes emigrated to developed countries, given their disagreements with Chavez’s radical reforms. The second phase corresponds to the end of the Latin American commodity growth. Venezuelan economy entered a great recession and migrant profile shifting, people from lower classes migrated to geographically proximate countries, such as Colombia. Finally, the third phase of migration began in 2015, after the election of Nicolas Maduro as president. The collapse of the Venezuelan economic leads to a severe humanitarian crisis, including a lack of food security and a broken public health system [6]. The current phase has to Peru as one of the principal migrant-receiving, with around one million Venezuelan migrants [7], significantly higher than internal migration flows [8]. Consequently, the Venezuelan migratory phenomenon is a unique and novel that deserves special attention and research.

The association between international migration and mental health is conditioned to several factors, and self-perceived discrimination may play a significant role. International migration has been discussed as a potential risk factor for mental health illness [9, 10]. Besides psychiatric illness, psychosocial factors may play a role in immigrants’ subjective wellbeing [9, 10]. Indeed, the literature suggests some plausible mechanisms to explain why there might be a higher prevalence of mental health illness in some vulnerable migrant groups [11–13]. Conflict and migration-related experiences, socioeconomic disadvantage, social marginalization, and a sense of sacrifice and sadness are critical elements for explaining this health disadvantage [12–15]. Evidence suggests that self-perceived discrimination among migrants profoundly affects their physical and psychological wellbeing in European and Asian countries [16–21]. In general, the relationship between the risk of mental illness and international migration continues to be complicated and difficult to disentangle. However, for Latin America, discrimination seems to be one of the main factors that could explain this problem.

Considering the above, this study aims to evaluate the association between self-perceived discrimination and mental health problems in the Venezuelan population living in Peru. The findings may help policymakers in a context where flows of Venezuelan migration may grow in coming years, as a complex humanitarian crisis is taking place in the region due to the largest Venezuelan exodus in history.

## Methods

### Design and data sources

We carried out a secondary cross-sectional analysis based on data from the 2018 Venezuelan Population Residing in Peru Survey (ENPOVE-2018), designed by the National Institute of Statistics and Informatics of Peru. The ENPOVE-2018 included a nationally representative urban sample of six Peruvian regions (Tumbes, La Libertad, Arequipa, Cusco, Lima, and Callao), which were selected since they reported the highest flows of Venezuelan migration. Likewise, the survey collected information on several dimensions of the Venezuelan population’s wellbeing, such as demographics, socioeconomic status, immigration status, discrimination, and violence characteristics. Data were collected from 26 November 2018 to 31 December 2018 through structured questionnaires applied by trained interviewers at home with every household member. The response rate was 99.6%.

### Population, sampling, and sample

The ENPOVE-2018 survey involved 3611 households and 9487 Venezuelans residing in Peru. This survey used a probabilistic, stratified, and region-independent sampling, where sampling units were blocks and households with Venezuelan residents. Our study included only the working-age population (aged 14+ years) with health indicators available, including mental health characteristics. We also excluded the population with missing labor-income values.

### Variables and measures

The outcome variable was the presence of mental health problems defined by the survey as adverse emotional and psychological reactions such as fear, anger, anxiety, or stress. This self-reported variable was built through a question with a binary answer (yes/no): “Since your arrival in Peru: Did you present with any problem associated with fearing, anger, anxiety, stress or similar?”

The main exposure was the self-perception of discrimination for being a Venezuelan immigrant. This self-reported variable was collected through a question with binary answer (yes/no): “Have you been discriminated against for Venezuelan immigrant status since your arrival in Peru?”

Moreover, we included the following control variables: sex (binary: male/female), age groups (ordinal in tertiles), marital status (binary: married or living together/other), minimum wage (binary: yes/no), education level (ordinal: no education/primary/secondary/tertiary), time spent in the country since arrival (ordinal: 6 months/6–12 months/+ 12 months in Peru), socioeconomic status constructed by INEI (ordinal: lower/middle/higher), and perceived social support (binary: yes/no).

### Statistical analysis

All analyses were performed in Stata 15.0® (StataCorp, College Station, Texas, US) considered sampling weights [22]. We describe numeric variables using mean + standard deviation and categorical variables using relative/absolute frequencies. We tested a preliminary association between self-perceived discrimination and mental health problems by Pearson’s Chi-Squared test. The same procedure was done to show the association between control and outcome variables.

To assess the association between self-perceived discrimination and mental health problems, we estimated prevalence odds ratios with their 95% confidence intervals (CI) using logistic regression models at crude (cOR) and adjusted (aOR) by statistical (*p*-value under 0.2 in bivariate analysis) and epidemiological criteria (according to the classic confounding definition) [23]. In adjusted models, the minimum wage was not included due to collinearity with socioeconomic status. Moreover, we estimated marginal effects to measure changes in the likelihood of mental health problems given self-perceived discrimination. We considered statistically significant coefficients for a *p* < 0.05.

Finally, we performed a propensity score matching (PSM) [24, 25] approach as a robustness check. PSM approach is a useful technique to minimize selection bias arising from observable variables. In our analysis, the PSM allowed us to compare discriminated and non-discriminated Venezuelan immigrants with similar characteristics. We used different matching algorithms [24, 26], namely nearest neighbor matching, five nearest neighbor matching, radius matching, and kernel matching. Several studies suggest using kernel matching as the main statistic estimator and the rest as a sensitivity analysis [27, 28].

## Results

### Sample characteristics

Of 9487 Venezuelans surveyed, 7939 were working-age population, and full information was available for 6806 of them, represented 85.7% of the working-age sample (Descriptive characteristics of the non-included sample are in A1 Table). From the final sample, 39% have perceived discrimination due to their migration status, and 6.3% reported at least one mental health problem related to fear, anger, anxiety or stress. The mean age was 30.8 ± 0.2, 58% were male, 57% were married or living with their partner, and around 85% completed at least a secondary educational level. The mean monthly salary-income was 1116 ± 14.4 soles (approximately US$329), 33% earn less than minimum wage, around 30% had spent at least one year in Peru, only 1.4% had received social support from any institution in the country; and 11.9, 55.3 and 32.7% were qualified in the lower, middle and higher socioeconomic status, respectively (Table 1).

**Table 1:** Characteristics of Venezuelans living in Peru

Characteristics	Sample size	Percentage^a^	95% CI
Mental Health problems			
No	6459	93.70	[91.86,95.14]
Yes	347	6.30	[4.86,8.14]
Perceived discrimination			
No	4232	60.97	[58.22,63.65]
Yes	2574	39.03	[36.35,41.78]
Sex			
Female	2883	42.66	[41.09,44.25]
Male	3923	57.34	[55.75,58.91]
Age groups			
14–26	2539	38.12	[36.06,40.22]
27–33	2117	29.98	[28.18,31.84]
34–78	2150	31.90	[29.89,33.98]
Age (Years)^b^		30.8 + 9.3	
Marital status			
Other	2906	42.87	[40.65,45.11]
Married or living with partner	3900	57.13	[54.89,59.35]
Educational level			
Basic	1032	14.82	[12.95,16.90]
Secondary	2913	43.27	[40.87,45.71]
Tertiary	2861	41.91	[39.64,44.21]
Minimum wage			
< minimum wage	2565	32.52	[30.41,34.71]
> minimum wage	4241	67.48	[65.29,69.59]
Monthly salary^b^		1116 + 538	
Time living in Peru			
6 months	2459	30.90	[28.66,33.24]
6–12 months	2969	44.83	[42.52,47.17]
+ 12 months	1378	24.26	[21.94,26.75]
Perceived social support			
No	6622	98.62	[98.10,99.00]
Yes	184	1.38	[1.00,1.90]
Socio-economic status			
lower	659	11.92	[8.31,16.82]
middle	3035	55.33	[49.12,61.39]
higher	3112	32.74	[27.42,38.55]

^a^ Percentage considered sampling weights

^b^ For numeric variables, percentage represent the mean value + standard deviation

### Differences by mental health problems

We found a significant association between mental health problems and self-perceived discrimination. Venezuelans who perceived being discriminated against showed a higher prevalence of mental health problems than their non-discriminated counterparts (*p* < 0.001). Moreover, we found a significant association between mental health problems and sex; female Venezuelans reported a higher prevalence of mental health problems than male counterparts (*p* < 0.001) (Table 2).

**Table 2:** Characteristics of Venezuelan immigrants by mental health condition

Characteristics	Non-reported mental health problems			Reported mental health problems			
	Sample size	Percentage^a^	95% CI	Sample size	Percentage^a^	95% CI	*p*-value
Perceived discrimination							
No	4080	62.39	[59.76,64.95]	152	39.88	[31.86,48.48]	*p* < 0.001
Yes	2379	37.61	[35.05,40.24]	195	60.12	[51.52,68.14]	
Sex							
Female	2687	41.50	[39.74,43.29]	196	59.93	[51.55,67.76]	*p* < 0.001
Male	3772	58.50	[56.71,60.26]	151	40.07	[32.24,48.45]	
Age groups							
14–26	2402	38.07	[35.89,40.30]	137	38.86	[32.85,45.24]	0.542
27–33	2021	30.19	[28.37,32.09]	96	26.78	[20.77,33.79]	
34–78	2036	31.73	[29.63,33.92]	114	34.35	[28.84,40.32]	
Marital status							
Other	2740	42.53	[40.24,44.85]	166	47.85	[40.99,54.79]	0.147
Married or living with partner	3719	57.47	[55.15,59.76]	181	52.15	[45.21,59.01]	
Educational level							
Basic	983	14.68	[12.79,16.79]	49	16.85	[12.29,22.65]	0.665
Secondary	2769	43.45	[41.01,45.93]	144	40.65	[32.10,49.80]	
Tertiary	2707	41.87	[39.55,44.22]	154	42.51	[34.72,50.69]	
Minimum wage							
< minimum wage	2441	32.56	[30.32,34.88]	124	31.95	[26.13,38.40]	0.859
> minimum wage	4018	67.44	[65.12,69.68]	223	68.05	[61.60,73.87]	
Time living in Peru							
6 months	2359	30.95	[28.70,33.29]	100	30.24	[22.47,39.34]	0.640
6–12 months	2802	44.61	[42.26,46.99]	167	48.05	[40.67,55.52]	
+ 12 months	1298	24.44	[22.04,27.00]	80	21.70	[16.25,28.36]	
Perceived social support							
No	6282	98.61	[98.06,99.00]	340	98.75	[96.73,99.53]	0.833
Yes	177	1.39	[1.00,1.94]	7	1.25	[0.47,3.27]	
Socio-economic status							
lower	643	12.47	[8.68,17.61]	16	3.74	[1.74,7.86]	0.054
middle	2872	54.76	[48.48,60.89]	163	63.87	[49.96,75.79]	
higher	2944	32.77	[27.38,38.65]	168	32.39	[20.98,46.35]	

^a^ Percentage considered sampling weights

### Association between perceived discrimination and mental health

In the unadjusted logistic regression model, Venezuelans who perceived being discriminated against had higher odds of reporting mental health problems than those who did not perceive discrimination (cOR:2.50, 95%CI:1.77–3.53). This result was similar in statistical (aOR:2.35, 95%CI:1.67–3.30) and epidemiological (aOR:2.39, 95%CI:1.69–3.37) adjusted models (Table 3). Other variables associated with mental health problems were sex and socioeconomic status. Findings from crude prevalence odds ratios are in A2 Table.

**Table 3:** Association between self-perceived discrimination and mental health problems

Characteristics	Sample size = 6806									
	Crude model			Adjusted model 1^a^			Adjusted model 2^b^			
	cOR	95% CI	*p*-value	aOR	95% CI	*p*-value	aOR	95% CI	margins	*p*-value
Perceived discrimination										
No	Ref.									
Yes	2.50	[1.77,3.53]	*p* < 0.001	2.35	[1.67,3.30]	*p* < 0.001	2.39	[1.69,3.37]	0.050	*p* < 0.001

cOR: crude odds ratio, aOR: adjusted odds ratios, margins: marginal effects

^a^ Adjusted model 1 included sex, marital status and socio-economic status

^b^ Adjusted model 2 included sex, age groups, marital status, educational level, time living in Peru, perceived social support and socio-economic status

The marginal effects showed that Venezuelans who perceived being discriminated against had a 5% higher probability of presenting a mental health problem compared to those who did not perceive discrimination (*p* < 0.001) (Table 3).

### Effect of perceived discrimination on mental health, propensity score matching

In our analysis, PSM models successfully reduced selection bias due to measured confounders from 5 to 1% in all cases approximately. On average, the final adjusted PSM models showed that Venezuelans who perceived being discriminated against increased in 3.5 percentage points their probability of presenting mental health problems compared to their non-discriminated counterparts (*p* < 0.01) (Table 4).

**Table 4:** Effects of self-perceived discrimination on mental health problems

Outcome	Matching algorithm	Coeff^a^	Bias before matching	Bias after matching	*p*-value
Mental health problems	kernel	0.038	4.887	1.627	*p* < 0.001
	nearest neighbor	0.039	4.887	0.646	*p* < 0.01
	5 nearest neighbor	0.033	4.887	1.604	*p* < 0.001
	radius	0.039	4.887	0.646	*p* < 0.01

Coeff: estimated coefficient

^a^ PSM were adjusted by sex, age groups, marital status, educational level, time living in Peru, perceived social support and socio-economic status

## Discussion

### Summary of main findings and international comparison

Our results show that self-perceived discrimination is associated with mental health problems among Venezuelans living in Peru, independent of relevant control variables. Migration is a core issue of global health in which multidisciplinary studies should analyze all phases of the process: before, during and after transit [1, 14, 29, 30]. There is a growing interest in developing research focused on post-migration stressors’ psychological effects in the settlement environment, where discrimination has a fundamental role [4, 10, 12]. Venezuelans face multiple challenges in living and surviving out of their country. Therefore, forced migration has significantly increased in the last two years [31, 32]. That has led to significant consequences on both Venezuela and Latin American countries, including the impacts on health [31, 33]. The current crisis in Venezuela and the subsequent migration is unprecedented in the region and Peru is a country that has received a significant proportion of Venezuelan migrants [7]. We found a higher prevalence and a higher probability of presenting mental health problems in Venezuelans with self-perceived discrimination compared to Venezuelans without this perception. Our study constitutes one of the first analysis of this association in Latin America and are relevant to the current geopolitical discussion. They could also be useful in international health policy decision-making processes related to international migration and population health issues.

Our findings are consistent with the literature regarding perceived discrimination, as previous studies found that it is associated with mental health alterations in migrant population. A meta-analytic review concluded that perceived discrimination’s pervasiveness has detrimental effects on psychological wellbeing [34]. A study of seven European countries reveal that the migrant population had a higher frequency of depressive symptoms and that discrimination was associated with this outcome [17]. In Norway, a study found that perceived discrimination was not associated with general health; however, it was associated with higher odds of mental health problems, even after controlling for socio-demographic and psychosocial variables [35]. In England, a study reported that discrimination experiences were associated with common mental disorders; however, magnitudes varied by migration status and race/ethnicity [18]. A Dominican Republic study suggested that perceived discrimination and humiliation contributed to Haitian migrant mental ill-health [19]. In Brazil, discrimination was associated with posttraumatic stress disorder, anxiety and depression symptoms in Haitian immigrants [16]. In summary, international evidence suggests that discrimination, as a social stressor, leads to mental health problems in migrant population. This association must be understood in a complex multifactorial context, even more in Latin-America [1, 9, 12].

The association between perceived discrimination and mental health problems could be influenced by different mechanisms, including social, psychological, and biological dimensions. First, discrimination has been identified as a post-migration stressor that negatively affects social integration and poor social integration contributes to a higher rate of inequality in mental health disorders [36, 37]. Secondly, acculturation stress increases mental health problems in immigrants and discrimination is an essential contributor to this problem [3, 38, 39]. Again, self-esteem has been recognized as an essential mediator over the effect of perceived discrimination on depression, anxiety and psychological wellbeing in migrants [5, 40]. Moreover, perceived discrimination has a direct effect on physical and mental health. Additionally, it indirectly affects stress response levels and health behaviors, conditioned by social support, stigma, and coping style [41]. Moreover, discrimination induced cortisol dysregulation, especially in immigrants and other racial/ethnic minorities [42, 43]. This psychobiological stress interacts with dopamine, serotonin and other neurotransmitters produce anxious and depressive symptoms [44]. Therefore, discrimination can lead to mental health problems via an intricate network of interrelated mechanisms.

### Implications of our findings to policy and practice

The “Venezuelan phenomenon” is complex and challenging. The humanitarian crisis has naturally caused a massive migratory exodus, with varying degrees of receptiveness to immigrants in different countries of the region [32]. According to international evidence available, international migration directly impacts health outcomes in a population and, in turn, on public health policy decisions in each locality [3]. This process has essential repercussions in Venezuela and neighboring receiving countries, affecting several areas: health, education, labor and others [31–33]. Discrimination has been recognized as a problem that the migrant population in different latitudes must deal with [4, 45]. Furthermore, deterioration in mental health is a severe issue among migrant populations [11–13]. Hence, discrimination and mental health are essential trace markers of the humanitarian crisis and constitute indicators of a complex interaction between strategies implemented by receiving countries, the reaction of the host society and the immigrants’ response.

Approximately one million Venezuelan migrants live in Peru, 88.8% are formal residents or regularization processes for resident status. Most of them constitute an essential flow of human resources (with a high proportion of them with a higher education level) that have been reinserted into the Peruvian labor force (mainly at technical and manual activities) [7]. Among migrant populations, integration strategies could improve their mental health while increasing the acceptability of diversity, reducing discrimination towards immigrants, thereby contributing to better mental health [35, 46]. Community-level interventions providing immigrants opportunities to increase social networking among members of the same country and those native-born in a host country may be helpful resources for improving mental health among immigrants [21, 47]. It is plausible that one who has a better understanding of the host culture might have inspired opportunities to participate in social activities with host country members, leading to an improved sense of belonging, which are significant predictors of better mental health. All these considerations should be taken by health and social authorities at the national level in countries such as Peru, which are receiving large influxes of migrants, especially from Venezuela.

### Limitations and strengths

Our study has several limitations. First, both mental health problems and perceived discrimination are self-reported, so selection and recall bias may affect outcomes reporting. Although there is controversy about measuring discrimination experiences, self-report is one valid way [48]. Second, we included in our analysis only factors identified in the ENPOVE-2018 survey. Other factors influence the association between perceived discrimination and mental health problems that we have not considered. Third, we do not have information about Venezuelans’ mental health problems before they arrived in Peru. Finally, with a cross-sectional survey, we cannot establish a causal effect of discrimination on mental health problems. Despite this, the main strengths of our study are that it is based on an urban nationally representative survey, and, to best of our knowledge, it is the first study that assesses the association between perceived discrimination and mental health problems in Venezuelan immigrants to Peru. Our findings are valuable for policymakers, public health and global health practitioners, as well as health professionals, given the structural and historical challenges of the Peruvian health system.

## Conclusions

There is evidence that perceived discrimination is associated with the deterioration of mental health in Venezuelan migrants living in Peru, a complex and under-researched topic. Research related to this problem constitutes a highly relevant tool for health and healthcare decision-making in Latin American countries. Study findings support the call for the urgent health protection of Venezuelan migrants in Peru. This population reports high degrees of self-perceived discrimination and is struggling to maintain their mental health. Future research should continue developing this topic, mainly through a more profound understanding of mental health needs and protective strategies for Venezuelan immigrants, using longitudinal approaches to achieve more robust estimates and understanding of underlying causal mechanisms.

## Supplementary Information

**Additional file 1: A1 Table.** Characteristics of the non-included sample. **A2 Table.** Unadjusted models for control variables.

## Data Availability

Our study used information from the public domain (http://iinei.inei.gob.pe/microdatos/).

## Abbreviations

ENPOVE: Venezuelan Population Residing in Peru Survey
CI: Confidence intervals
cOR: Crude odds ratios
aOR: Adjusted odds ratios
PSM: Propensity Score Matching
